# Community Mobilization to Guide the Public Health Response During the 2022 Ontario Mpox Outbreak: A Brief Report

**DOI:** 10.1093/ofid/ofae195

**Published:** 2024-10-15

**Authors:** Darrell H S Tan, Adam Awad, Austin Zygmunt, James Murray, Daniel Warshafsky, Sharmistha Mishra, Dane Griffiths

**Affiliations:** Division of Infectious Diseases, St Michael's Hospital, Toronto, Ontario, Canada; MAP Centre for Urban Health Solutions, St Michael's Hospital, Toronto, Ontario, Canada; Department of Medicine, University of Toronto, Toronto, Ontario, Canada; Gay Men's Sexual Health Alliance, Toronto, Ontario, Canada; Max Ottawa Community Health, Ottawa, Ontario, Canada; Department of Health Protection, Public Health Ontario, Toronto, Ontario, Canada; Department of Family Medicine, University of Ottawa, Ottawa, Ontario, Canada; HIV and Hepatitis C Programs, Ontario Ministry of Health, Toronto, Ontario, Canada; Office of the Chief Medical Officer of Health, Public Health, Ontario Ministry of Health, Toronto, Ontario, Canada; Division of Infectious Diseases, St Michael's Hospital, Toronto, Ontario, Canada; MAP Centre for Urban Health Solutions, St Michael's Hospital, Toronto, Ontario, Canada; Department of Medicine, University of Toronto, Toronto, Ontario, Canada; Gay Men's Sexual Health Alliance, Toronto, Ontario, Canada; Toronto Public Health, Toronto, Ontario, Canada

**Keywords:** communications, community engagement, epidemic response, monkeypox, mpox

## Abstract

The 2022 mpox epidemic predominantly affected gay, bisexual, and other men who have sex with men (GBM). Led by a provincial community program and co-galvanized by clinician-researchers, GBM community leaders in Ontario coordinated a robust response, representing a reproducible strategy for community engagement and mobilization during future epidemics.

Ontario, Canada, was among the first jurisdictions to experience a major outbreak when the global mpox epidemic began in mid-2022 [1]. The province's first case was reported on 20 May 2022, and Ontario declared an mpox outbreak the following day. Case counts rapidly escalated and peaked in mid-July 2022 [2]. There were 692 confirmed cases by the time the outbreak was declared over on 10 December 2022 [3], mostly among adult gay, bisexual, and other men who have sex with men (GBM). Community engagement was a key element of Ontario's epidemic response, led by the Gay Men's Sexual Health Alliance (GMSH). Provincial public health authorities rapidly identified GMSH as a leader in sexual health promotion for GBM and funded it to help coordinate a community-engaged response and provide a focused mpox awareness campaign. These efforts were particularly urgent because Toronto Pride celebrations were scheduled for late June 2022 and would be the first to be held in person since 2019 due to coronavirus disease 2019 (COVID-19). We describe key enablers and outcomes of this multifaceted community response.

## COMMUNITY MOBILIZATION

GMSH is a provincial program that promotes sexual health by producing digital content, campaigns and resources and collaborates with community-based human immunodeficiency virus (HIV) service organizations to increase the capacity of sexual health services for Ontario GBM. In mid-April 2022, GMSH staff were closely monitoring the emergence of mpox following sex events in Europe, and on 19 May 2022, GMSH issued its first community alert. Within days, it began convening weekly online community mobilization meetings, including GBM leaders, clinician-researchers, and public health stakeholders, to share information about the evolving outbreak and facilitate collaboration.

These meetings created an invaluable forum for the community to provide strategic advice to provincial and local public health leaders on communicating their own health and vaccine promotion strategies to diverse sexual and gender minority individuals, including those living with HIV and racialized individuals, in a culturally competent fashion and using appropriate language. Public health authorities appreciated the efficiencies that resulted from GMSH's leadership and the opportunity to directly engage with community members for feedback on outbreak management and immunization campaigns [4]. The partnership also enabled public health to focus on vaccine administration, case management, and contact tracing while GMSH provided tailored, community-facing messaging.

A critical enabler of the effective community mobilization during the mpox response in Ontario was the existing local network of community-based organizations already active in the HIV response and in GBM sexual health. Ontario has a longstanding history of grassroots, community-led work in advocacy, policy making, service delivery, and research. The shared values of this network and its leaders—including sex positivity, harm reduction, and an antistigma and antidiscrimination lens, together with a history of engagement with other evidence-based health interventions, such as HIV preexposure prophylaxis—facilitated meaningful collaboration.

## COMMUNICATION CAMPAIGN

**Figure 1.**
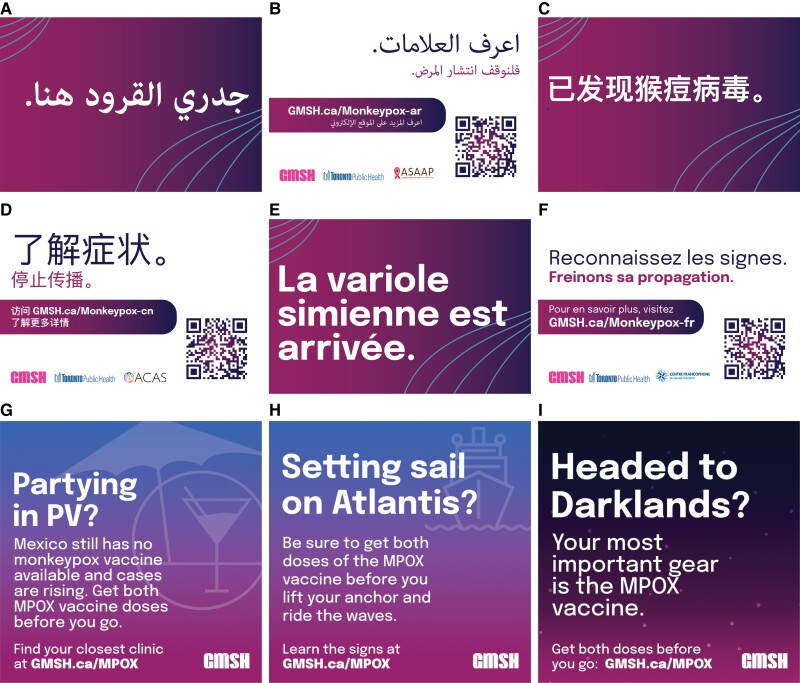
*A–F,* The mpox awareness campaign conducted by the Gay Men's Sexual Health Alliance (GMSH) included posters, postcards and digital materials in 10 languages regarding mpox symptoms and prevention strategies, including vaccination. *G–I,* Materials used candid language and focused messaging to reach communities at risk, including those traveling internationally to queer festivals for sex.

A core GMSH activity was its comprehensive multilingual awareness campaign, which focused on mpox symptoms and prevention strategies, including vaccination (Figure 1). The campaign used a sex-positive approach to reach communities of GBM at risk. Outputs included a central website with information in 10 languages featuring vaccine clinic locations and research links; print resources for patients and clinicians; and publicity materials like posters, postcards, and large banners for pride festivals. A suite of digital ads was promoted on popular geotargeted sexual networking applications and websites province wide, and video content featured popular artists and influencers (eg, drag queens). Materials used candid language (atypical for governmental awareness campaigns) and focused on at-risk scenarios (eg, international travel to queer festivals for sex [Figure 1]. On average, each person reached through the paid advertising campaign would have seen mpox messaging 20 times. Ads were delivered more than 74 million times, resulting in 380 000 clicks, indicating substantial engagement.

Nuances regarding some GBM sexual practices and networks were emphasized to health partners; for instance, public health officials were prompted to expand their attention to “sex-on-premises venues” within the queer community, rather than narrowly focusing on bathhouses. Staff representing such venues also became part of the network's weekly mobilization meetings, providing front-line insights about the perspectives and concerns of their staff and clientele.

## PROMOTING VACCINATION

Ontario deployed a focused immunization campaign using a third generation live attenuated, nonreplicating Modified Vaccinia Ankara–Bavarian Nordic smallpox vaccine (Imvamune), as recommended by Canada's National Advisory Committee on Immunization [5]. Mass immunization clinics used during the COVID-19 pandemic were repurposed to offer vaccine to eligible populations [6]. The first mass immunization clinic was held in Toronto, Ontario, on 12 June 2022, and, in total, 37 470 doses were administered to 35 545 people in Ontario between 4 May and 24 October 2022 [7].

Community expertise was harnessed and integrated when developing vaccine policy. Rollout of the mpox vaccine in the most affected Ontario cities exemplified calls elsewhere for “decentralized, community-based and culturally and contextually aligned service-delivery models that meaningfully engaged community leaders in designing and implementing local vaccination campaigns” [8]. Federal, provincial, and local public health officials attended Ontario's community mobilization meetings but did so as guests, consulting the community on vaccine eligibility criteria, language to be used in policy documents, and implementation plans. Public health units partnered with community to design and implement pop-up vaccine clinics in locations selected and/or hosted by local organizations, including bathhouses [9].

Issues of critical importance to the community included that clients not be deemed vaccine ineligible or turned away by vaccine clinics for seemingly failing to meet clinical criteria and that staff not force clients to disclose their HIV status in public clinics, despite immunocompromised HIV-positive individuals being prioritized for vaccination in policy documents. Recognizing the potential for stigma and privacy concerns to limit disclosure of same-sex behavior and HIV status to clinicians [10], as well as the intersecting contexts of institutionalized racism and other forms of discrimination in healthcare, community members advocated that any client presenting to vaccine clinics be immunized, an approach that was largely followed in Ontario throughout the campaign [9].

## ADVOCACY

An important community initiative was an advocacy campaign directed to key federal cabinet ministers, seeking financial supports for individuals forced to self-isolate due to mpox. Given mpox's lengthy duration of 2–4 weeks [11, 12], people's livelihoods could be significantly affected while they were following public health directives [13]. A community open letter, endorsed by 66 organizations from across Canada but conceived during the Ontario community meetings, advocated for emergency financial supports [14]. Although the campaign did not ultimately achieve these goals, it galvanized a broad-based movement among stakeholders, and it was positively received by community members, who had spoken out about the financial strains associated with strict mpox isolation measures [15].

## RESEARCH

The community response also had positive outcomes on research. Community priorities were incorporated into local mpox studies, by including isolation-related financial hardships and mpox-related stigma among their outcome measures [16], and were reflected in knowledge translation outputs from local work. For instance, early during the epidemic, community representatives protested the Western media's excessive reliance on imagery of mpox lesions on black skin, joining the Kenya-based Foreign Press Association Africa, which had argued that such stereotypes “assign calamity to the African race and privilege or immunity to other races” [17]. Highlighting this concern during a community mobilization meeting prompted Canadian clinician-researchers to develop image libraries featuring local cases [18] and to disseminate international resources depicting mpox lesions on a variety of skin tones [19]. These actions echoed further calls from within the field of dermatology to promote health equity by advocating for inclusive language and imagery in the mpox response, while engaging in conversation about the stigmatizing nature of visible skin disease [20].

## CONCLUSIONS

The collaboration among the Ministry of Health (as funders and policy makers) and GMSH facilitated a community-informed response to Ontario's mpox outbreak that was timely, credible, and effective, with several important lessons. First, rapid community mobilization and engagement have multiple practical benefits for epidemic response, by bolstering the legitimacy of public health messaging during a crisis, engendering an efficient “division of labor” with health specialists, and facilitating community engagement in research. Second, providing adequate funding for community engagement is critical. From a community perspective, the ability to work more closely with government and public health partners enabled the overall response to mpox to be more accessible and impactful for GBM at risk of mpox. Importantly, many community-based organizations in the field of sexual health remain chronically underfunded, jeopardizing their very sustainability and the feasibility of true community-engaged epidemic preparedness in the long term.

Third, apprehensions about minimizing stigma are important and are best addressed through active engagement. Parallels between mpox and the early days of the HIV pandemic were striking—both were harmfully perceived as “gay diseases,” could “out” people through their cutaneous disease manifestations, and were initially characterized by unknowns that provoked discrimination and fear. For some, there was apprehension about focusing too exclusively on GBM for these reasons and even about referring to mpox as a sexually transmitted infection. Paradoxically, however, well-founded fears of stigmatizing queer communities can also engender harm: echoing the wisdom of a generation of HIV/AIDS activists, “silence equals death.” The Ontario experience during the mpox outbreak response demonstrates the importance of leadership, engagement, and mobilization within the community itself and of governmental and public health stakeholders privileging these perspectives when codeveloping interventions.
